# Women in epidemiology

**DOI:** 10.1017/ash.2022.284

**Published:** 2022-08-10

**Authors:** Suzanne F. Bradley

**Affiliations:** 1 Division of Infectious Diseases, Department of Internal Medicine, University of Michigan Medical School, Ann Arbor, Michigan; 2 Infectious Diseases Section, Veterans Affairs Ann Arbor Healthcare System, Ann Arbor, Michigan

## What motivated you to pursue a career in healthcare epidemiology and/or antimicrobial stewardship?

Like many women, my career path was not a linear one. I came of age in the early 1970s, *Ms.* magazine, and Gloria Steinem. I grew up in a family that was involved in health care, but I was its first physician. Successful matriculation in medical school was not a given, and I was assured that if I failed, I could always pursue a traditional job for women such as teacher, librarian, nurse, or secretary. My physician role models were men and family friends who had private practices; I knew little about academia. Unbeknownst to me, one of the most prominent hematologist-oncologists in my community lived next door, but she was known only as “mom” by one of my childhood playmates.

Although I generally excelled academically in college, I had difficulty with organic chemistry. I could not grasp the teaching style of my first professor and had to drop the course. Fortunately, I had an outstanding teacher in summer school and did very well. During medical school at Buffalo and my internal medicine residency at Ohio State, I developed an interest in infectious diseases (ID) through interactions with role models, again virtually all men. As a group, I found ID physicians to be very welcoming and I was in awe of their ability to solve diagnostic dilemmas. In short, I wanted to be like them and enrolled in a fellowship at the University of Michigan.

I turned down the opportunity to pursue healthcare epidemiology twice. At one interview, I thought the infection control track would be too narrow and I wanted to explore the wide variety of scientific opportunities that a career in ID had to offer. Later, as a junior faculty member at the University of Michigan, I was offered the infection control job. However, I had just been awarded a National Institutes of Health (NIH) grant that addressed the impact of malnutrition on mechanisms of fever in a murine model, and I had been repeatedly told that it was important to be productive and to focus on a narrow area of research that I could call mine, if I were to succeed in academia. I recognized how busy a hospital epidemiologist would be at a large tertiary medical center. The clinical track did not provide many opportunities for advancement, and I had little leverage to bargain for protected research time, additional personnel, or extra monetary support, so I did not pursue this opportunity further.

Over time, I realized that the career advice I was given might be true early in one’s career but did not necessarily apply later. Just as there have been researchers whose narrow areas of investigation reached a dead end and who, without alternatives, needed to switch jobs, there have also been successful individuals who kept a broader view, adapted, and recognized when new and more fruitful opportunities presented themselves. Being prepared broadly for new opportunities, serendipity, and a willingness to take risks allowed me to switch my career trajectory on several occasions.

## Tell us about the role of mentorship in shaping your career; describe a pivotal mentor relationship either within your institution or within SHEA that altered the trajectory of your career

While at Michigan, I was fortunate to meet with Carol Kauffman who ultimately became a lifelong mentor and friend. Although some faculty members were pursuing topics that were “hotter” at the time, I chose Dr Kauffman primarily because she was someone of intelligence and integrity that I wanted to emulate. Through her, I began a long collaboration initially focused on the impact of aging, nutrition, and immunocompromise on the host response to infection in murine models, and I met many others who served as role models including Thomas Yoshikawa. I gained much experience in the application of murine models of infection, temperature measurement, macrophage biology, measurement of cytokines, and molecular methodology. Dr Yoshikawa appointed me as Section Editor of the Biosciences Section of the *Journal of the American Geriatrics Society*. My broad knowledge of scientific methods acquired while working in basic science was invaluable when I reviewed and made decisions about papers that were frequently unrelated to infectious diseases.

In the early 1980s, Dr Kauffman’s laboratory shifted focus to the study of the epidemiology of methicillin-resistant *Staphylococcus aureus*. Studies involving nursing homes and antibiotic resistance paralleled my interest in aging and infection, and thus, began my involvement in healthcare epidemiology. I began to attend the Society for Healthcare Epidemiology of America (SHEA) annual meetings. At one reception, I met Donald Craven, who suggested that I apply to join the SHEA Long-Term Care Committee (LTCC). I contacted Lindsay Nicolle and was accepted as a member, which was a seminal event for me. Dr. Nicolle ensured that younger members were allowed to propose and lead the development of manuscripts about topics related to nursing home infection. I ultimately succeeded her as Chair of the LTCC and was involved in oversight and publication of several articles covering topics such as influenza and tuberculosis, which were cited on the Centers for Disease Control and Prevention website for many years. Through these activities, I interacted with many members of the SHEA board including presidents Michael Tapper, John Boyce, and Trish Perl. Ultimately, I was nominated for and elected Academic Councilor. During my tenure on the board, a search was being conducted for editorship of *Infection Control & Hospital Epidemiology* (ICHE). Given my editorial experience, I was asked to apply, and to my great surprise, I was selected! Several years later, the position of hospital epidemiologist at my Veterans Affairs Healthcare System became available. I convinced the leadership that I was the best candidate for the job and, for the first time, some salary support was provided.

## What were some notable barriers in your career?

At the time, Michigan did not hire its trainees as faculty except under extenuating circumstances. Today, my colleagues can expect to be hired at a competitive starting salary as well as additional funds to set up their research efforts in anticipation of future independent funding. I had a 3-year mentored NIH grant that paid $25,000 salary and $5,000 for supplies. There was no hard-money job, and the chair encouraged me to look elsewhere because I would not be invited to stay. It was not clear what other program could mentor me and provide the support and expertise in geriatrics and ID necessary to complete my work. Dr Kauffman was one of very few senior women in the department, and she was able and willing to back me with money for supplies and by supplementing my faculty salary. I was able to take a brief financial risk in the hope of longer-term gain, and this approach paid off.

Several years later, geriatric medicine had “hard-money” salaried positions but few faculty. My research background, coupled with a year-long geriatrics fellowship, led to a successful 38-year career. I am one of few physicians who has formal clinical and research training in geriatrics and ID. My expertise in ID was hard won, and I resolved to keep my options open and remain clinically active and competent in this discipline despite the extra time involved. This decision was fortuitous when my geriatrics clinical load increased; ID was looking for faculty, and I joined them full-time.

The criteria for promotion have not always been as transparent as they are now. We tend to believe that if you work hard, you will succeed in your profession and be recognized for your achievements. Research productivity, leadership capabilities, titles, election to academic societies, and professional recognition are highly valued aspects of academic advancement; however, these opportunities were rarely granted to women in the past. For example, when I arrived in 1984, there were only 2 female full professors and few women achieved tenure. Before seeking promotion, I had sought opportunities to lead at my institution, but the usual response was that there were only so many positions to go around. I recall the embarrassment at a divisional meeting when our chief acknowledged the achievements of every division member but there were none he could recount when it was my turn.

I recognized that if opportunities were not available at the institutional level, then I would have to seek them elsewhere. I ultimately was promoted to professor at Michigan, due in part to opportunities for leadership and professional recognition from SHEA and other professional societies. As a result, I have continued to help guide my junior colleagues through the promotion process and recommend them for leadership and professional recognition opportunities. I am pleased that change has come to my institution and that women are no longer sitting on the sidelines in the cheering section but are being recognized for their accomplishments.

It is not surprising that I encountered challenges over my 15 years as Editor of ICHE. Initially, I had to gain the trust of the editorial board; at the first meeting several members were vocal in their lack of support and some resigned. I was not well known as a member of the infection control and public health communities, nor had I graduated with a degree in epidemiology. Research in my area of nursing home–related infections was only beginning to gain traction in the infection prevention literature. I was fortunate to have the advice and support of my team of journal editors and publishers, who helped navigate the politics involved. We had a small staff, and most of the day-to-day work was done by 2 people, the managing editor and me. After 5 years, the decision to change publishers was very time-consuming, and we risked losing momentum with the change in personnel and workflow; I had developed enough experience as Editor to adjust to those changes. A bigger challenge was the unexpected loss of our permanent managing editor. Delays and disruption of the review process had the potential to anger authors and greatly reduce the likelihood that they would submit their work to us. Fortunately, I was able to expedite decisions thanks to the enormous efforts of the journal editors and the editorial board, who took on more reviews and turned them around quickly.

I am particularly proud that our ICHE team delivered 15 volumes of high-quality and relevant infection control content during my tenure—we never missed a deadline. Articles were reviewed rapidly, and the time to publication online and in print were greatly reduced. We published the first supplemental issues in ICHE including the Compendia, Decennial Meeting, Agency for Healthcare Research, and compilations of articles with various themes from antimicrobial stewardship to environmental disinfection. The ICHE website was updated, and virtual collections of important articles about popular topics and podcasts about recent manuscripts were released. Every aspect of the journal was overhauled, including the cover. Press releases and Twitter feeds became the norm with the assistance of the publications committee, managing editor, and SHEA Journal Club. In 2021, the impact factor of ICHE was the highest to date!

Although it is important for leaders to delegate responsibilities so they can focus on the bigger picture, it was unusual that I had sufficient staff or resources. I had to be intimately involved in day-to-day aspects of research, the journal, or infection control at my institution. The pandemic was perhaps the biggest challenge of my career. Amy Lyons, my sole infection control practitioner (ICP), was tireless, and we worked side by side without interruption while my clinical and journal duties continued. The upside was that we were finally granted permission to hire a second ICP trainee after years of waiting. I was particularly honored and touched to receive the Michigan Medicine Department of Internal Medicine Lifetime Achievement Award in 2022 and the 2021 VA Ann Arbor Clinician of the Year Award.

## What were the biggest drivers of your success?

In a word, persistence and the belief that I could do a job even if I was told “no” were key. This does not mean that my faith was blind. Like many women, I clearly had “imposter syndrome,” constantly reassessing whether I was qualified or good enough. My response to this challenge was typical—I always overprepared, and I still do!

There are many ways to attain a goal, but some approaches did not make sense in the context of my experience. For example, I was often asked about my 5-year plan. At the time, I had few role models and little reassurance that I could be a successful academic physician and researcher, let alone have any long-term aspirations of leadership. Is there anything wrong with having a 5-year plan? In a word, no, but it requires constant and honest reflection and realistic assessment of your road map along the way. Why do you have the goal? Do you continue to have the energy, drive, and enthusiasm to pursue that goal? What if your goals change? Don’t be blind to other opportunities as they arise.

Traditionally in medicine, new trainees are told to identify an area of medical research that is unique and that evolves into a track record of independent and lifelong investigation. In my view, this approach ignores the fact that many infection control investigations are increasingly collaborative, and independence is often established by a mentor who merely removes their name from papers. Few investigators truly work alone anymore. A narrow focus also does not guarantee lifelong research success. In our field, where new and emerging infections have been identified all too frequently, it is not uncommon that successful investigators have shifted their research focus. Many new observations emerge from the clinical arena, so it is important to maintain those skills and remain alert to opportunities.

Several colleagues encouraged me to take advantage of every opportunity, so when new career choices arose, I was prepared. For example, research in aging enabled my career in geriatrics and my work in the epidemiology of infection in nursing homes. Without journal experience in geriatrics, I would not have been prepared to take on the editorship of ICHE. Without experience in epidemiology research, I would not have been prepared to lead an infection control program and the hospital’s pandemic effort.

## What career advice would you share with young professionals?

There is no one correct career path. As one of my mentors said, “Some folks will seem to have it easier than others; let it go and move on. For example, some colleagues know influential people have an impressive pedigree. Those factors may get their foot in the door, but they may not stay there.” Work in areas that you enjoy and with mentors who will look after your interests beyond theirs. Be prepared to reinvent yourself many times during your career. Narrow topics that are popular today may not stay that way; maintaining a broader scope of interests can be beneficial. In the words of a department chair, “Be kind to your clinical colleagues who will take you in when your research is not going well [sic].” Be sure that you have enough protected time, personnel, and resources to do the job.

Above all, keep your eyes open because opportunities may come from various sources. For example, I regret not knowing my grandfather better. He loved fixing things and renovating old houses, and that is what we talked about. He didn’t talk about his work in the early days of bacteriology and public health in Massachusetts. Once, he asked me if we had to worry about the spread of infection with all the antibiotics available? Or did I know about Tommy Francis, an old colleague of his who spearheaded the national trial of the Salk vaccine? What discussions we would have had if I had only followed up with more questions. Maybe I would have gravitated to infection control or public health sooner. A missed opportunity, indeed!

## What books or essays most influenced you?

In my spare time, I enjoy reading about history and travel, and I enjoy mystery novels. I find it informative to see how others faced challenges and solved them. My current postretirement read is by David J. Shulkin, *It Shouldn’t Be This Hard to Serve Your Government: Our Broken Government and the Plight of Veterans*. Other favorite autobiographies and biographies have included Madeline Albright, Katherine Graham, Alexander Hamilton, Eleanor Roosevelt, and Jane Goodall. When trying to problem solve, I tend to start by doing research. Finding a seminal article, no matter how old, is very satisfying. My colleagues’ historical perspectives have also been invaluable professionally. To paraphrase Bob Weinstein, who once responded to my concerns about asking a prominent author to cut their lengthy manuscript, “Everyone thinks their words are important. If someone refuses to cut their paper, send them a copy of the scientific article that changed the world [sic].” Enclosed was a copy of a one and one-half page article by Watson & Crick!

